# Assessment of noise levels in the intensive care unit using Apple Watch

**DOI:** 10.1186/s13054-020-02852-3

**Published:** 2020-04-06

**Authors:** Tommaso Scquizzato, Arianna Gazzato, Giovanni Landoni, Alberto Zangrillo

**Affiliations:** 1 Department of Anesthesia and Intensive Care, IRCCS San Raffaele Scientific Institute, Milan, Italy; 2 Faculty of Medicine, Vita-Salute San Raffaele University, Milan, Italy

Alarms from monitors, medical devices and staff activities increase noise levels in the Intensive Care Unit (ICU) and may disrupt sleep patterns [1] contributing to the development of delirium and post-intensive care syndrome [2]. The World Health Organization (WHO) recommend that hospital noise levels should not exceed 35 A-weighted decibels (dBA) during the day and 30 dBA at night [3]. However, daytime noise levels in ICU were found of around 60 dBA [4]. Apple Watch (Series 4 and 5) takes advantage of the internal microphone to regularly sample sound levels in the environment and might play a role in monitoring noise in the ICU.

We investigated the feasibility of analyzing data from an Apple Watch to measure noise levels in the ICU. Accordingly, we exported Health data from the personal Apple Watch of a nurse working in a 14 beds referral cardiothoracic ICU managing patients after cardiac surgery and those with cardiogenic shock, refractory cardiac arrest, and respiratory failure. Noise levels were compared between daytime (7 a.m. - 11 p.m.) and night-time (11 p.m. - 7 a.m.). Data extraction and statistical analysis were performed with the “Pandas” Python Library. An open-source Jupyter notebook has been made available together with this publication on GitHub (https://github.com/tscquizzato/ICU-Noise-Levels-Apple-Watch) with a step-by-step guide to repeat our experience.

Consecutive 1086 samples measured during 48 shifts (48% from 7 a.m. to 7:30 p.m. and 52% from 7:15 p.m. to 7:15 a.m.) between November 1, 2019 and February 29, 2020 were extracted. The average sound level was 66 ± 6.1 dBA (Fig. 1). Sound levels significantly differed between daytime and night-time (67 ± 6.7 dBA vs. 64 ± 4.2 dBA, *p* < 0.001) (Fig. 2). The highest sound level was 89 dBA and was recorded on Monday between 12 a.m. and 1 p.m. The lowest one was 31 dBA between 3 p.m. and 4 p.m. on Sunday. In only 2.8% of samples, noise levels during daytime were below 35 dBA. During the night, sound levels were always above 30 dBA.

**Fig. 1:**
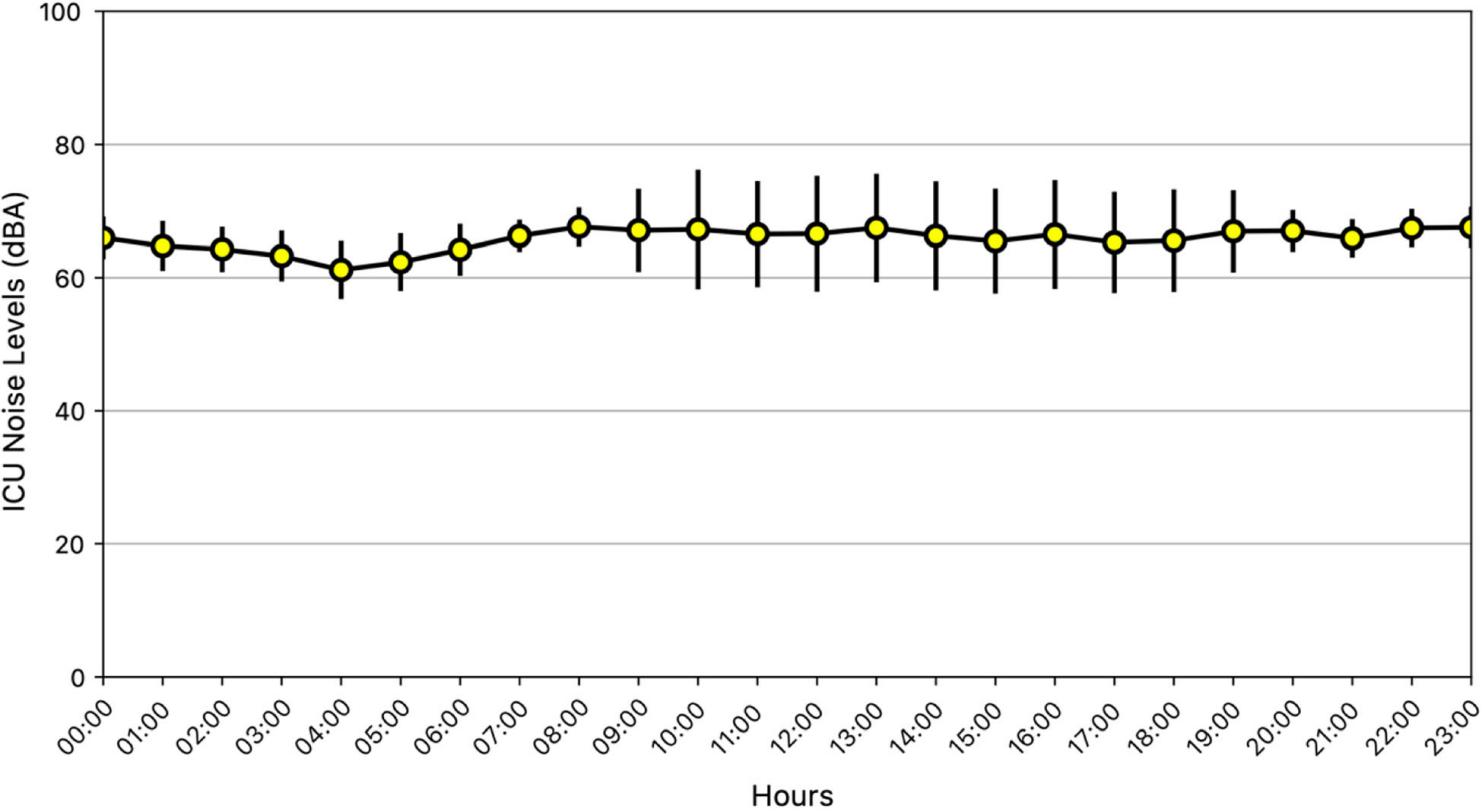
Trend of average sound pressure levels over the 24 h. Values in A-weighted decibels (dBA) are expressed as mean with standard deviation

**Fig. 2:**
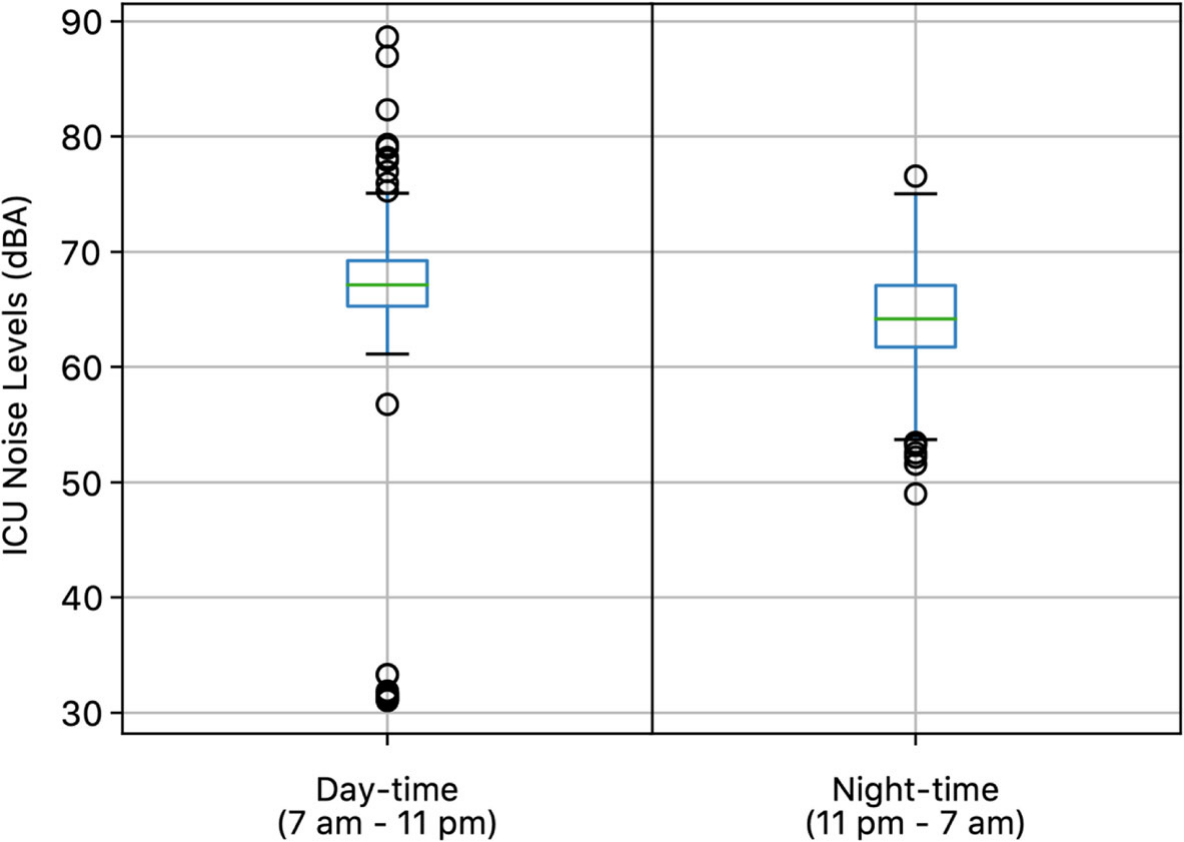
Boxplot comparing sound pressure levels during daytime (7 am – 11 pm) and night-time (11 pm – 7 am)

The analysis of noise levels in the ICU using an Apple Watch is feasible and easy to perform. Overall, noise levels were almost always above the recommended values, consistent with previously published studies [4]. The role of wearable devices to measure noise levels deserves to be further investigated. Such devices might also be worn by patients to accurately quantify noise levels and compare with sleep quality and recovery.

## Data Availability

Not applicable.

## Abbreviations

ICU: Intensive care unit
dBA: A-weighted decibels
WHO: World Health Organization
